# Effect of atrial high-rate episodes (AHREs) on functional status and quality of life (QoL) in heart failure—cardiac resynchronization therapy population

**DOI:** 10.1186/s43044-025-00613-7

**Published:** 2025-02-03

**Authors:** Lamyaa Elsayed Allam, Youssef Abdel Moneim, Hayam Mohammad Eldamanhoury, Sherif Mohammad Aziz Eltoukhy

**Affiliations:** Cardiology Department, Ain Shams University, Cairo, Egypt

**Keywords:** Heart failure with reduced ejection fraction, Cardiac resynchronization therapy, Atrial high-rate episodes, 6-min walk test, Minnesota living with heart failure questionnaire

## Abstract

**Background:**

New type of arrhythmia called atrial high-rate episodes (AHREs) has been discovered thanks to the ability of cardiac electronic implantable devices to track, record, and analyze complex arrhythmias. The aim is to determine factors associated with AHRE in HFrEF/CRT patients and the effect of AHRE on functional capacity and quality of life (QoL).

**Results:**

We interrogated 100 patients' devices to gauge the incidence and burden of AHRE, then assessed their functional capacity using the standard 6-min walk test (6MWT), and evaluated their QoL using the Minnesota Living with HF questionnaire (MLHFQ) score. 34% of patients had AHRE, and 91.2% of them had AF. By multivariate logistic regression analysis, smoking (OR 9.426, 95% CI [1.33, 66.65], *P* 0.025), higher BMI (OR 1.336, 95% CI [1.09, 1.635], *P* 0.005), and increased LAVI (OR 1.16, 95% CI [1.063, 1.262], *P* < 0.001) are independent predictors for AHRE. There was a significant correlation between AHRE and the distance walked during 6MWT when compared to the distance expected for an equivalent healthy individual (82.02 ± 17.22% in the non-AHRE group vs. 75.15 ± 15.78% in the AHRE group, *P* < 0.001). It was found that AHRE was statistically linked to a higher total MLHFQ score (46.76 ± 9.82 in the AHRE group vs. 36.97 ± 7.76 in the non-AHRE group, *P* 0.032), with higher physical scores in the AHRE group.

**Conclusion:**

AHRE significantly reduces functional status and perceived quality of life in HFrEF patients receiving CRT. Longer than five minutes of AHRE was associated with a higher MLHFQ score and worse performance on the 6MWT. In that patient population, smoking, obesity, and elevated LAVI were independent predictors of AHRE.

## Background

Cardiac implantable electronic devices (CIEDs) have advanced significantly during the last few decades. Currently, these devices can treat bradycardia with pacemakers (PM), ventricular arrhythmias with implanted cardioverter defibrillators (ICDs), and HF in patients who meet the medical criteria for cardiac resynchronization therapy (CRT). With one or more intracavitary leads, CIEDs can now analyze, record, and potentially treat various arrhythmia types. Moreover, the atrial lead enables the recording of atrial high-rate episodes (AHREs), arrhythmic episodes characterized by a high atrial rate [1].

Continuous cardiac monitoring technologies, such as CIEDs, can identify subclinical and asymptomatic atrial tachyarrhythmia, which often happens before the onset of clinical atrial fibrillation (AF) [2, 3].

Previous studies have demonstrated that AHREs are strongly correlated with clinically diagnosed AF [4] and are independently linked with an increased risk of ischemic stroke and systemic embolism [5–7].

Researchers have undertaken several initiatives to evaluate the risk factors that lead to AHRE development [8] and to assess the association between AHREs and clinical outcomes in patients with CIEDs such as stroke, cardiovascular events, and all-cause mortality [9, 10].

Few researchers have focused on the relationship between AHRE and the functional capacity and quality of life of HF patients. So, this study aims to determine the burden and predictors of AHRE in HFrEF patients receiving CRT and the effect of AHRE on the patient's functional capacity and quality of life.

## Methods

### Study population

This prospective study recruited 100 consecutive patients who had HErEF and received CRT-P or CRT-D for at least 6 months before the study at a large-volume tertiary care facility in Egypt from June 2023 to June 2024.

We included patients who met the guidelines' requirements for CRT and successfully had their devices implanted [11].

The ethics committee of Ain Shams University approved the research before its initiation.

An MS 518/2023 approval number is provided. Written consent was obtained from each participant, ensuring proper confidentiality and privacy.

Patients who had interstitial pulmonary fibrosis, bronchial asthma, cerebrovascular disease, skeletomuscular abnormalities, severe chronic kidney disease (CKD) (glomerular filtration rate (GFR) < 15 mL/min/1.73 m^2^ for 3 months or more, regardless of its cause) [12], permanent AF, previous history of clinical AF or flutter, or pacemaker dependency due to heart block were not allowed to take part in the study.

Regular CRT device interrogation was used to evaluate the composite primary outcome prediction. For a year, the patients were routinely examined in the clinic every two months in order to gather data.

### Assessment of incidence and burden of AHRE

For the analysis of AHRE incidence, automated mode switch (AMS) episodes retrieved from device diagnostics were collected and analyzed.

To mitigate the risk of overdiagnosing based on the EHRA consensus statement [13], along with the guidelines of the ESC [14], an AHRE should be considered when a device enters AMS mode and the atrial rate is equal to or exceeds 175 beats/min (bpm), and this condition persists for a minimum duration of 5 min as identified by the atrial lead.

In this study, a cutoff of > 5 min was utilized to exclude overdiagnosis of several atrial signals, including sinus tachycardia and far-field over-sensing, to detect AHRE [4]. The other atrial noises were ruled by examining the intracardiac ECGs recorded in the pacemakers as much as possible [15].

The following data of AHRE were collected: rate, rhythm, frequency per month, duration of the longest episode, and cumulative duration of all episodes per patient.

### Assessment of echocardiographic data

Every patient had transthoracic echocardiography using a GE Healthcare Vivid S5 equipped with a 3 MHZ transducer, in addition to machine-integrated electrocardiogram recording. Each patient's left atrial and ventricular volumes, including the left ventricular ejection fraction (LVEF), left ventricular end-systolic volume (LVESV), left ventricular end-diastolic volume (LVEDV), and left atrial volume index (LAVI), were measured.

Simpson's approach was used to average the volumes acquired from the 2-chamber and 4-chamber views, and the ejection fraction (EF) was calculated based on the established American Society of Echocardiography protocols [16].

### Assessment of functional capacity and quality of life

We used Minnesota Living with Heart Failure Questionnaire (MLHFQ) score, 6-min walk test (6MWT) distance, and New York Heart Association (NYHA) functional class for this assessment.

**The 6-min walk test (6MWT)** is a submaximal exercise test applied to assess the functional status of HF patients. It was done according to the American Thoracic Society [17].

The study took place along a long, straight, level, enclosed corridor with a solid surface. A 30-m walking corridor was used, with markers on the wall every 3 m.

To complete one lap at 60 m, the patient had to turn around at the end of every thirty meters.

A chair at the end of the hallway marked the turnaround point, while tape marked the beginning and end of each 60-m lap on the floor.

The values were compared to the anticipated distance walked by a normal person of the same age and height using the calculations published by Enright et al. [18] and validated by Casanova et al. [19] because pre-CRT implantation data were not available.

**Minnesota Living with Heart Failure Questionnaire (MLHFQ)** is a self-administered disease-specific questionnaire made specifically for heart failure patients. It has 21 items with six-point Likert scales that reflect the different impacts of HF on health-related quality of life (HRQoL), ranging from 0 (none) to 5 (very much). The total score can be calculated from 0 to 105, which represents the best to worst QoL. Additionally, two dimensions are measured on the questionnaire: physical (8 items, 0–40) and emotional (5 items, range 0–25). Only the final eight items—out of a total of 21—are used in calculating the final score [8].

The Arabic version of the MLHFQ has been verified and translated [9]. Cutoffs were established at 24 to indicate a good quality of life, 24 to 45 a moderate quality of life, and > 45 a poor quality of life. Studies have demonstrated a correlation between these cutoffs and 6MWT, NYHA functional class, and survival status [20, 21].

### New York heart association (NYHA) functional class

A popular and straightforward method for classifying individuals with heart failure into one of four groups based on the severity of their symptoms at rest and during activity is the NYHA Classification system. This method uses classes I through IV, where class I denotes less severity and higher numerals denote greater severity [22].

## Statistical analysis

The statistical software for social sciences, version 23.0 (SPSS Inc., Chicago, Illinois, USA), was used to analyze the recorded data. When the distribution of the quantitative data was parametric (normal), they were displayed as ranges and as mean ± standard deviation. In contrast, nonparametric data, or variables having non-normal distributions, were displayed as the median with the interquartile range (IQR). Qualitative variables appeared as numbers and percentages. Both the ***Shapiro–Wilk and Kolmogorov–Smirnov tests*** were used to examine the normality of the data. ***Independent-sample t test of significance*** was employed when comparing between two means. ***Fisher's exact test*** was used instead of the Chi-square test for comparing groups with qualitative data when the expected count in any cell was less than 5. ***Multivariate logistic regression analysis:*** Odds ratios (OR) with 95% confidence intervals were used to assess the overall association between each potential risk and the incidence of CRT non-response and AHRE. ***The confidence interval was 95%, and the accepted error margin was*** 5%. So, the p value was considered significant if n ≤ 0.05.

## Results

### Baseline characteristics of the study population

The demographic data for each of the 100 consecutive HFrEF/CRT patients are shown in Table 1*.*

**Table 1:** Baseline characteristics of the study population

Baseline characteristics	Study population (no.100)
Age (Yrs) (Mean ± SD)	60.02 ± 7.18
Gender: Male, *n* (%)	81/100 (81%)
BMI (Kg/m^2^):	
Mean ± SD Normal range (BMI 18.5– < 25) Overweight (BMI 25– < 30) Obese (BMI > 30)	30.00 ± 6.40 17 (17.0%) 44 (44.0%) 39 (39.0%)
Risk factors and comorbidities:	
DM, *n* (%) Hypertension, *n* (%) Dyslipidemia, *n* (%) Smoking, *n* (%) Alcoholic, *n* (%) OSA, *n* (%)	71/100 (71%) 81/100 (81%) 68/100 (68%) 31/100 (31%) 2/100 (2%) 8/100 (8%)
Ischemic etiology	64 (64%)
CHA_2_DS_2_‐VASc score	
Range Mean ± SD Median (IQR)	2–6 3.59 ± 1.03 4 (3–4)
Drug treatment:	
ARB/ARNI, ARB, ACEi β blockers, *n* (%) Loop diuretics, *n* (%) MRAs, *n* (%) SGLT2 inhibitor, *n* (%) Empagliflozin Dapagliflozin	100/100 (100%) 100 /100 (100%) 10/100 (10%) 98/100 (98%) 100/100 (100%) 19 /100(19%) 81/100(81%)
CRT-P	55 (55%)
Echocardiographic data	
LV EF % (Mean ± SD) LV EDV (ml) (Mean ± SD) LV ESV (ml) (Mean ± SD) LAD (mm) (Mean ± SD) LAVI ml/m^2^(Mean ± SD)	35.18 ± 4.99 282.57 ± 41.49 183.19 ± 38.30 41.42 ± 5.85 38.95 ± 9.89
Degree of mitral regurgitation (MR)	
Mild Moderate Severe	77 (77%) 22 (22%) 1 (1%)
RVSP (Mean ± SD)	47.56 ± 10.92

*BMI* body mass index (kilogram/meter^2^); *DM* diabetes mellitus; *OSA* obstructive sleep apnea; *ARB* angiotensin II receptor blockers; *ACEi* angiotensin-converting enzymes inhibitors. *ARNi* angiotensin receptor neprilysin inhibitor, *MRAs* aldosterone receptor antagonists; *SGLT2 inhibitor* sodium-glucose co-transporter 2 inhibitors; *CRT-P* cardiac resynchronization therapy pacemaker; *LV EF* left ventricular systolic ejection fraction; *LV EDV* left ventricular end-diastolic volume; *LVESV* left ventricular end-systolic dimension; *LAD* left atrial dimension; *LAVI* Left atrial volume index; *ml* milliliter; *mm* millimeter; *RVSP* right ventricular systolic pressure

CHA_2_DS_2_‐VASc score included congestive heart failure, hypertension, age ≥ 75 years, diabetes mellitus, stroke or transient ischemic attack, vascular disease, age 65–74 years, sex category

### Quantification of AHRE burden

Thirty-four out of 100 patients (34%) suffered from at least one episode of AHRE. Thirty-one patients (31% of the study population and 91% of AHRE patients) displayed irregular atrial rhythm during tachycardia, indicating atrial fibrillation episodes, while 3 patients (3% of the study population and 9% of AHRE patients) experienced episodes of regular atrial rhythm. The median frequency per month was 3.5 episodes with an IQR of 2.75-6 (Table 2).

**Table 2:** Atrial high-rate episodes (AHRE) burden data of the studied patients

AHRE data	Study population (no.100)
Patients with AHRE	34 out of 100 (34%)
Rhythm	
Irregular (AF) Regular (AT)	31 (91.2%) 3 (8.8%)
Rate of AHRE (BPM)	
Mean ± SD Median(IQR)	201.17 ± 22.6 199 (180–210)
Frequency per month	
Mean ± SD Median (IQR)	5.67 ± 5.4 3.5 (2.75–6)
Duration of the longest AHRE episodes (min)	
Mean ± SD Median (IQR)	6.6 ± 1.3 6.2 (5.5–7.5)
Cumulative AHRE duration per patient (min)	
Mean ± SD Median (IQR)	18.23 ± 50.08 7(5.75–8.5)

*AHRE* atrial high-rate episode; *AF* atrial fibrillation; *AT* atrial tachycardia; *BPM* beats per minute; *min* minutes

### Functional capacity and quality of life of the study population

Regarding 6MWT results, the mean distance walked in meters was 446.87 ± 63.18, and the mean distance expected for healthy individuals of the same age, sex, and height was 561.27 ± 42.72. In comparison, the mean percentage of the distance walked was 79.68% ± 9.86% with a range of 52–91%; 28 patients (28.0%) had walked less than 80% of the distance expected for a healthy equivalent (Table 3).

**Table 3:** 6MWT results among the study population

	Study population (*n* = 100)
*Distance walked in meters*	
Range Mean ± SD	280–562 446.87 ± 63.18
*Distance expected for a healthy individual (in meters)*	
Range Mean ± SD	454–674 561.27 ± 42.72
*Percentage (%) of distance walked in comparison to expected for a healthy individual*	
Range Mean ± SD < 80% ≥ 80%	52%-91% 79.68% ± 9.86% 28 (28%) 72 (72%)

The mean total MLHFQ score was 40.30 ± 21.69, with a mean physical score of 18.08 ± 9.40 and a mean emotional score of 12.69 ± 6.03. According to the total MLHFQ score, 36 patients (36.0%) had a score of > 45 indicating a poor QoL, 31 patients (31.0%) had a score of 24–45 indicating a moderate QoL, while 33 patients (33.0%) had a total MLHFQ score of < 24 indicating a good QoL (Table 4).

**Table 4:** MLHFQ results and NYHA classification among the study population

	Study population (*n* = 100)
*Physical dimension score*	
Range Mean ± SD Median (IQR)	2–42 18.08 ± 9.40 14.0 (9.3–20.8)
*Emotional dimension score*	
Range Mean ± SD Median (IQR)	2–23 12.69 ± 6.03 14.5 (7–17)
*MLHFQ Total score*	
Range Mean ± SD Median (IQR)	9–87 40.30 ± 21.69 38 (20–51)
*MLHFQ Total score cutoffs*	
> 45 Poor QoL 24–45 Moderate QoL < 24 Good QoL	36 (36.0%) 31 (31.0%) 33 (33.0%)
NYHA class	No. of patients (% of total population)
Class I Class II Class III Class IV	33 (33%) 45 (45%) 22 (22%) 0 (0%)

By NYHA classification, 33% of patients had class I, and 45% had class III (Table 4).

### Risk factors associated with AHRE

By comparison between patients who suffered from AHRE versus non-AHRE group, there was a statistically significant higher prevalence of smoking in the AHRE group (50%) versus non-AHRE group (21%), *P* value 0.003. in addition to a higher BMI (28.14 ± 5.91in non-AHRE vs. 33.62 ± 7.06 in AHRE, *P* value < 0.001) (Table 5).

**Table 5:** Comparison between the non-AHRE group and AHRE group regarding baseline characteristics and echocardiographic parameters

	AHRE group (no.34)	Non-AHRE group (no.66)	Test value	*p* value	Sig
Age (years)	58.06 ± 12.19	61.03 ± 12.82	1.988	0.050	NS
Gender ( Male)	29 (85.3%)	52 (78.8%)	0.617	0.432	NS
Hypertension	24 (70.6%)	57 (86.4%)	3.629	0.057	NS
DM	28 (82.4%)	43 (65.2%)	3.225	0.073	NS
BMI	33.62 ± 7.06	28.14 ± 5.91	− 4.424	< 0.001	HS
Smoking	17 (50.0%)	14 (21.2%)	8.694	0.003	S
Ischemic etiology	24 (70.6%)	39 (59.1%)	1.273	0.259	NS
CHA_2_DS_2_‐VASc score	3.59 ± 0.75	3.59 ± 0.75	0.012	0.990	NS
QRS morphology (LBBB)	27 (79.4%)	48 (72.7%)	0.535	0.465	NS
Echocardiographic parameters LV ESV (ml) LV EDV (ml) LAVI	203.74 ± 42.78 304.44 ± 63.93 55.12 ± 11.57	172.61 ± 36.25 271.30 ± 56.97 30.62 ± 6.43	− 4.154 − 4.070 − 8.512	< 0.001 < 0.001 < 0.001	HS HS HS

*BMI* body mass index (kilogram/meter^2^); *DM* diabetes mellitus; *LBBB* left bundle branch block; *LVEDV* left ventricular end-diastolic volume; *LVESDV* left ventricular end-systolic volume; *LAVI* left atrial volume index.; *ml* milliliter; *mm* millimeter; *NS* non-significant; *S* significant; *HS* highly significant

According to echocardiographic data, the AHRE group had a significantly larger LV ESV (203.74 ± 42.78 ml vs. 172.61 ± 36.25 ml in non-AHRE, *P* < 0.001); significantly larger LV EDV (304.44 ± 63.93 ml vs. 271.30 ± 56.97 ml in non-AHRE, *P* < 0.001), and significantly higher LAVI, (55.12 ± 11.57 ml/m^2^ in AHRE vs.30.62 ± 6.43 ml/m^2^ in non-AHRE, *P* < 0.001) (Table 5).

By multivariate logistic regression analysis, it was found that smoking (OR 9.426, 95% CI [1.33, 66.65], *P* Value 0.025), higher BMI (OR 1.336, 95% CI [1.09, 1.635], *P* value 0.005), and increased LAVI (OR 1.16, 95% CI [1.063, 1.262], *P* Value < 0.001) are independent predictors for AHRE in the study population (Table 6).

**Table 6:** Multivariate logistic regression analysis for predictors of occurrence of AHRE

Parameters	*Β*	Wald	Sig	OR	95% C.I	
					Lower	Upper
Gender	− 1.546	0.904	0.342	0.213	0.009	5.164
Age	− 0.143	2.303	0.129	0.867	0.721	1.043
Hypertension	− 1.211	0.763	0.382	0.298	0.020	4.510
DM	0.030	0.001	0.984	1.030	0.059	18.054
BMI	0.290	7.894	0.005	1.336	1.092	1.635
Smoking	2.243	5.053	0.025	9.426	1.333	66.654
Etiology of the heart failure	0.972	0.442	0.506	2.644	0.150	46.556
CHA_2_DS_2_‐VASc score	1.050	0.810	0.368	2.857	0.291	28.087
QRS morphology	− 1.287	0.764	0.382	0.276	0.015	4.946
LV EDV	0.011	0.126	0.723	1.011	0.950	1.077
LV ESV	0.033	0.785	0.376	1.034	0.960	1.113
LAVI	0.150	12.527	< 0.001	1.161	1.069	1.262

*BMI* body mass index (kilogram/meter^2^); *DM* diabetes mellitus; *LVEDV* left ventricular end-diastolic volume; *LVESV* left ventricular end-systolic volume; *LAVI* left atrial volume index; *ml* milliliter; *mm* millimeter; *OR* odds ratio; *CI* confidence interval

### Impact of AHRE on the quality of life and functional capacity:

**Fig. 1:**
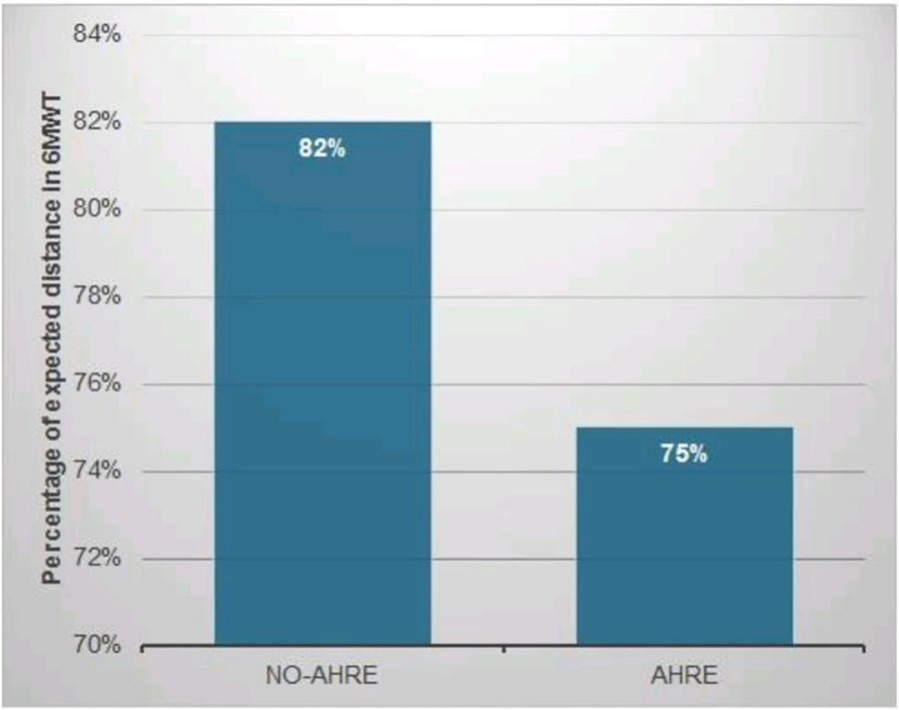
Comparison between non-AHRE and AHRE groups regarding the percentage of expected distance walked for a healthy individual

Regarding 6MWT, there was a significant difference between both groups regarding distance expected for an equivalent healthy individual (82.02 ± 17.22% in the non-AHRE group vs. 75.15 ± 15.78% in the AHRE group, *P* value < 0.001), as the number of individuals who achieved less than 80% of the expected distance between the two groups was 17 out of 34 patients (50%) in the AHRE group versus 11 out of 66 patients (16.7%) in non-AHRE group, *P* value < 0.001 (Table 7 and Fig. 1).

**Table 7:** Comparison between non-AHRE and AHRE groups regarding 6MWT and MLHFQ

Parameters	AHRE group (no.34)	Non-AHRE group (no.66)	Test value	*p* value	Sig
6MWT distance walked in meters	430.09 ± 90.32	455.52 ± 95.66	1.933	0.056	NS
% of expected distance walked for a healthy individual	75.15 ± 15.78	82.02 ± 17.22	3.482	< 0.001	S
Individuals who achieved < 80% of the expected distance Individuals who achieved ≥ 80% of the expected distance	17 (50.0%) 17 (50.0%)	11 (16.7%) 55 (83.3%)	12.368	< 0.001	HS
MLHFQ physical score	18.38 ± 3.86	13.77 ± 3.10	− 1.831	0.05	S
MLHFQ emotional	14.12 ± 2.96	11.95 ± 2.51	− 1.715	0.090	NS
MLHFQ total score	36.97 ± 7.76	46.76 ± 9.82	− 2.179	0.032	S
MLHFQ total score interpretation					
> 45 Poor QoL 24–45 Moderate QoL < 24 Good QoL	17 (50.0%) 7 (20.6%) 23 (34.8%)	19 (28.8%) 24 (36.4%) 10 (29.4%)	4.807	0.090	NS

*6MWT* 6-min walk test; *MLHFQ* Minnesota living with heart failure questionnaire; *QoL* quality of life; *NS* non-significant; *S* significant; *HS* highly significant

**Fig. 2:**
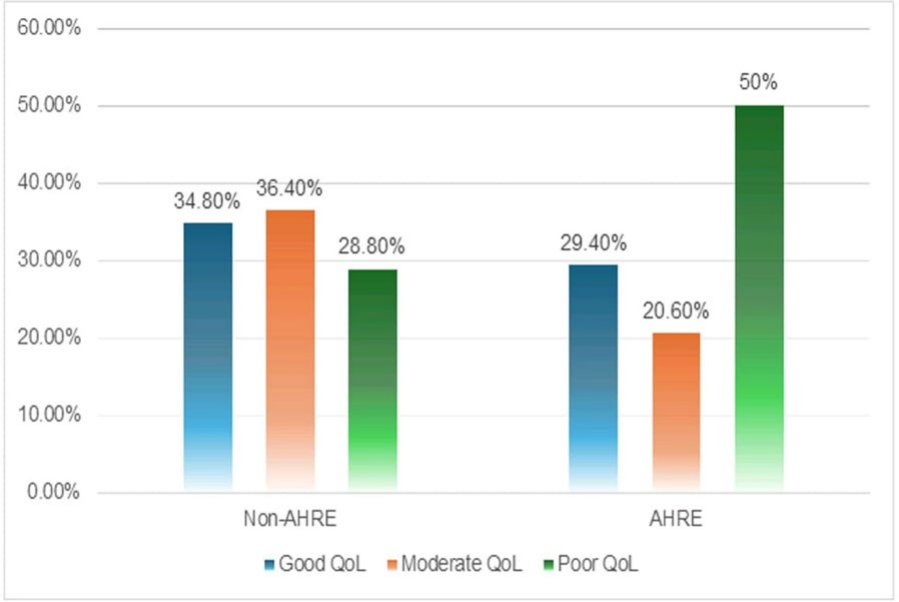
Comparison between non-AHRE and AHRE groups regarding QoL

As regards the quality of life (MLHFQ), there were statistically significant higher MLHFQ scores among the AHRE group (36.97 ± 7.76) versus the non-AHRE group (46.76 ± 9.82) with a *p* value of 0.032) and this is also associated with higher physical scores (18.38 ± 3.86 in AHRE group versus in non-AHRE group, *P* value 0.05) (Table 7 and Fig. 2).

## Discussion

Multiple studies of various types of pacemakers found a high prevalence of AHREs, but their clinical significance has remained a question. This study aimed to assess the factors associated with AHRE in HFrEF patients receiving CRT, as well as the impact of AHRE on functional capacity and quality of life.

In this study, smoking, obesity, and dilated left atrium are independent predictors for AHRE in the study population. In the 6-min walk test, there was a statistically significant difference between the AHRE and non-AHRE groups in the distance that a healthy person of the same size should be able to walk. Also, more people in the AHRE group failed to walk at least 80% of the expected distance. Regarding quality of life (MLHFQ), there were statistically significant lower MLHFQ scores among the non-AHRE group compared to the AHRE group, indicating that patients in the AHRE group had a lower quality of life.

The study relied on device diagnostics for AHRE detection, strictly selecting AHR episodes based on the current EHRA consensus statement [13]. As mentioned in the methodology section, 34% of the study population suffered from the AHRE and AF was responsible for it in 91.1% of those patients. These results were similar to earlier studies like Jędrzejczyk-Patej E et al. [3].

Based on the established criteria of AHRE, they discovered that device-performed atrial arrhythmia identification was accurate in about 90% of cases and that in 98% of patients with adequately diagnosed arrhythmia, atrial fibrillation (either alone or in conjunction with concurrent atrial flutter) was the cause of AHR episodes. The most common cause of inadequate detection in eleven percent of the subjects was ventricular far-field sensing in the atrial channel, a condition our patients did not exhibit.

### Risk factors associated with AHRE

Smoking and higher BMI were more prominent in the AHRE group of the study, in addition to larger LAVI and larger LV systolic and diastolic volumes. However only smoking, obesity, and increased LAVI were independent predictors of AHRE based on multivariate logistic regression analysis.

A few studies have examined the risk factors associated with AHREs lasting longer than 5 or 6 min [23, 24]. The literature has well established the correlation between smoking and obesity with atrial tachyarrhythmia, particularly with atrial fibrillation. In the ARIC study by Alanna et al. [25], the adjusted hazard ratios (HRs) for AF based on multiple variables were 1.32 (95% CI 1.10–1.57) for ex-smokers, 2.05 (95% CI 1.71–2.47) for smokers who are currently smoking, and 1.58 (95% CI 1.35–1.85) for smokers who have ever smoked. In contrast to people with normal BMI, Wang et al. showed adjusted hazard ratios for AF related to obesity of 1.46 (95% CI 1.03–2.07; *P* = 0.03) for women and 1.52 (95% CI 1.09–2.13; *P* = 0.02) for males [26].

Increased LA volume can be due to atrial cardiomyopathy which is defined as any complex of structural, architectural, contractile, or electrophysiological changes affecting the atria with the potential to produce clinically relevant manifestations based on the consensus statement published by Goette et al. [27]. Due to numerous pathogenic variables that cause atrial remodeling, such as elevated LA pressure, reactive lipid mediators of oxidative stress, and inflammatory signaling, atrial cardiomyopathy is frequently linked to cardiomyopathies and heart failure [27, 28].

There is a growing body of evidence suggesting that AF or AHREs are phenotypic expressions of atrial cardiomyopathy [29, 30]. According to Doundoulakis et al. [31], AHRE and AF may act as a marker of atrial cardiomyopathy which may increases the risk of stroke in comparison to general population.

Zhaodi et al. [23] discovered that in patients with atrioventricular block, a larger LA dimension predicted newly diagnosed AF (HR1.06; CI 1.01–1.14; *p* 0.046) following dual chamber pacemaker implantation.

In a different study, Li YG et al. [8] discovered that patients with sustained AHRE in CEIDs had larger LA diameter, LA volume, and LA volume index (*P* < 0.05, respectively). They also had significantly higher CHA2DS2‐VASc scores, with 95.5% (*n* = 42) of patients having a score of ≥ 2 (men) or ≥ 3 (women), as opposed to 78.3% of patients (*n* = 357) without AHREs (*P* = 0.007). However, there were no variations in the CHA2DS2‐VASc scores between the two groups in the current study. The rationale behind this is the study's inclusion of patients with HFrEF with CRT, whereas all patients with various cardiac implanted devices were included in the LiYG research. For the study sample, the presence of HF alone contributes one point to the CHA2DS2-VASc score, indicating a high initial score.

### Impact of AHRE on QoL and functional capacity

In this study, there was no significant correlation between the presence of AHRE and distance walking during 6MWT. As a standard, we calculated the expected distance walked by a normal individual of the same age, sex, weight, and height for each study subject [18, 19].

When expressing the results of the 6MWD test as a percentage of the expected distance, there was a highly statistically significant difference between the AHRE group and the non-AHRE group. There was also a significant difference between the two groups regarding the number of individuals who achieved less than 80% of the expected distance, indicating a substantial impact of AHRE on the functional capacity of the HF/CRT population.

As regards the quality of life, there was a statistically significant correlation between the prevalence of AHRE and the total MLHFQ score. The AHRE group was found to have higher scores compared to the non-AHRE group, indicating poorer QoL. This can be explained by the significantly higher physical scores in the AHRE group, while there was no statistically significant difference in emotional scores among both groups.

The physical dimension of MLHFQ addresses difficulties doing things with friends or family, sleeping difficulties, resting during the day, difficulty in working around the house, difficulty in being away from home, fatigue, and difficulty in walking or climbing stairs, while the emotional dimension deals with issues like feeling like a burden to friends or family, losing control, having trouble focusing or remembering things, feeling unhappy, and worrying [20].

Most of the studies assessed the impact of AHRE on clinical outcomes regarding stroke cardiovascular events and even all-cause mortality, but based on our knowledge, no studies evaluated its effect on functional capacity and quality of life in patients with heart failure.

According to research by Nishinarita R et al., the three main univariate predictors of worsening HF in patients with CEIDs were serum creatinine, presence of AHRE, and previous congestive HF. The multivariate Cox regression model identified the presence of AHREs (HR 1.27, 95% CI 1.11–1.5, *P* = 0.004), prior congestive HF (hazard ratio [HR] 6.7, 95% confidence interval [CI] 1.73–30.9, *P* = 0.006), and serum Cr (HR 3.82, 95% CI 1.74–8.4, *P* = 0.002) as independent predictors of worsening HF. In the multivariate analysis, when comparing the three groups (none, low, and high AHRE burden), the greatest independent predictors for worsening HF were the existence of AHREs and AHRE burden [9].

Evidence that AHRE progression raises overall mortality in ICD and CRT-D participants has been presented by Jiang et al. [10]. AHREs and overall mortality have previously been connected. Glotzer et al. [5] found that PPM recipients with AHREs had a higher death rate than those without (HR 2.48, 95% CI 1.25–4.91, *p* = 0.0092).

## Limitations

The sample size can be considered a limitation that may affect the generalizability of the findings. In addition, it is a single-center study. Another limitation is the collection of AHRE data during device checks rather than through home remote monitoring, which may result in missed episodes.

## Conclusions

AHRE significantly reduces functional status and perceived quality of life in HFrEF patients receiving CRT. Longer than five minutes of AHRE was associated with a higher MLHFQ score and worse performance on the 6MWT. In that patient population, smoking, obesity, and elevated LAVI were independent risk factors of AHRE.

## Data Availability

No datasets were generated or analyzed during the current study.

## Abbreviations

6MWT: Six-minute walk test
ACEI: Angiotensin-converting enzyme inhibitors
AF: Atrial fibrillation
AHRE: Atrial high-rate episode
AMS: Automated mode switch
ARNI: Angiotensin receptor neprilysin inhibitors
ARB: Angiotensin II receptor blocker
AT: Atrial tachycardia
BMI: Body mass index
BPM: Beats per minute
CHA_2_DS_2_‐VASc score: Included congestive heart failure, hypertension, age ≥ 75 years, diabetes mellitus, stroke or transient ischemic attack, vascular disease, age 65–74 years, sex category
CIEDs: Cardiac implantable electronic devices
CKD: Chronic kidney disease
CRT: Cardiac resynchronization therapy
DM: Diabetes mellitus
ECG: Electrocardiography
EHRA: European heart rhythm association
ESC: European Society of Cardiology
HFrEF: Heart failure with reduced ejection fraction
ICD: Implantable cardioverter defibrillator
GFR: Glomerular filtration rate
LAVI: Left atrial volume index
LBBB: Left bundle branch block
LV EF: Left ventricular ejection fraction
LVEDV: Left ventricular end-diastolic volume
LVESV: Left ventricular end-systolic volume
min: Minutes
ml: Milliliter
MLHFQ: Minnesota living with heart failure questionnaire
mm: Millimeter
NYHA: New York Heart Association
OSA: Obstructive sleep apnea
PM: Pacemakers
QoL: Quality of life
RBBB: Right bundle branch block
RVSP: Right ventricular systolic pressure
